# Development of an Ultra-Performance Liquid Chromatography-Tandem Mass Spectrometry Method for Hydroxylated Polychlorinated Biphenyls in Animal-Derived Food

**DOI:** 10.3390/molecules27217247

**Published:** 2022-10-25

**Authors:** Wenyu Zhao, Na Cui, Yuan Huang, Zihao Wang, Peilong Wang, Haijun Liang, Ruiguo Wang, Xiaoou Su

**Affiliations:** 1 Institute of Quality Standards and Testing Technology for Agro-Products, Chinese Academy of Agricultural Sciences, Beijing 100081, China; 2 CHINA FEED Magazine Agency, Beijing 100710, China

**Keywords:** hydroxylated polychlorinated biphenyls, food, determination, metabolite, UPLC–MS/MS

## Abstract

Hydroxylated polychlorinated biphenyls (OH-PCBs) are a group of metabolites biotransformed from polychlorinated biphenyls by animals with higher toxicities than their parent compounds. The present work developed and validated an analytical method for determinating penta-, hexa-, and hepta-chlorine substituted OH-PCBs in animal-derived food based on ultra-performance liquid chromatography–tandem mass spectrometry (UPLC–MS/MS) with isotope-dilution. The target analytes were extracted with a 50% n-hexane/dichloromethane (*v*/*v*), purified by sulfuric acid-silica gel, and separated by 5% hydrated silica gel, achieving a final concentration of 100 times before injection to LC–MS/MS. The limits of detection (LOD) and quantification (LOQ) for target OH-PCBs were within the ranges of 0.003–0.010 μg/kg and 0.009–0.030 μg/kg, respectively. Average recoveries ranged between 76.7% and 116.5%, with relative standard deviations of less than 18.4%. The proposed method is simple, time-saving, sensitive, and accurate, making it a powerful tool for risk monitoring of OH-PCBs in animal-derived food.

## 1. Introduction

Polychlorinated biphenyls (PCBs) were designated as one of the first twelve persistent organic pollutants (POPs) and have been governed by the Stockholm Convention since 2001 due to their characteristics of environmental mobility, persistence, bioaccumulative potential, and toxicity to living organisms [1]. PCBs are a group of 209 chemical compounds characterized by a typical structure of a biphenyl framework with from one to ten chlorine atoms attached [2]. They were manufactured as industrial chemicals for decades before the ban on production and use was adopted in the 1970s [3]. Nevertheless, PCBs have been found ubiquitously in the various environmental matrices and biosphere still [4,5]. PCB contamination in the food chain is often along with dioxins and other POPs with stronger toxicity [2]. This combined pollution in feed led to an incident involving a massive of PCB/dioxin-contaminated eggs in Belgium in 1999 and ultimately caused a global food crisis [6]. According to the investigation, about 90–98% of the average human exposure to PCBs resulted from dietary intake of animal-derived food [7]. Therefore, as the predominant source, animal origin food has received considerable attention for the pollution of PCBs.

Although PCBs are resistant to degradation, animals can metabolize some PCBs with specific structures into hydroxylated polychlorinated biphenyls (OH-PCBs). The extent and rate of PCB metabolism depend on their structural properties and the animal species. Those PCBs with vicinal non-chlorine substituted positions, especially in the meta- and para-positions of the biphenyl core, are more susceptible to cytochrome P450-mediated transformation [4]. OH-PCBs have a hydroxyl group on the molecular structure, thereby enhancing its hydrophilicity and similarity in some hormones (such as estrogen and thyroid hormones), revealing more endocrine-disrupting effects than their parent compounds [8]. There are 837 possible OH-PCB congeners in theory, whereas only several structures with five or more chlorines (penta- to hepta-chlorinated OH-PCBs) are commonly found in humans [4,9] and animals [10,11,12]. Low-chlorinated (mono- to tetra-chlorinated) OH-PCBs are usually expected to be subject to excretion from the bodies, so they are occasionally found in animal tissues [13].

The predominant OH-PCB congeners, for example, include 4-OH-CB187 and 4-OH-CB107 (mean level: 0.4, 0.1 ng/mL wet weight in sera) in American puerpera [9], 4-OH-CB187 and 4-OH-CB146 (20, 8.03 ng/g wet weight in livers) in arctic foxes [10], 4-OH-CB146 and 4-OH-CB187 (0.36, 0.21 ng/g wet weight in the blood) in Baikal seals [11], and 4-OH-CB107 and 4-OH-CB162 (0.13, 0.06 ng/g wet weight in the blood) in pigs from waste dumping site in South India [12]. In general, OH-PCBs are ready to be excreted via urine and bile in animals. However, some OH-PCBs can be stably retained in animal bodies and transferred from the maternal to the eggs, resulting in a secondary foodborne risk together with their precursor compounds [14]. Therefore, it is necessary to develop methods for the detection of OH-PCBs, especially the predominant congeners, in animal-derived food.

The detection of OH-PCBs is a great challenge because of its very low concentrations (ranging from low femto- to nanogram per gram) and the tremendous number of congeners with wide variation in physicochemical properties in samples. Therefore, it requires a very sensitive and selective instrument method with a thorough clean-up procedure. Gas chromatography (GC) with different detectors, such as mass spectrometry (MS) [15,16,17], high-resolution mass spectrometry (HRMS)[16], and electron capture detector (ECD)[18,19], are commonly employed to determine OH-PCBs in biological matrices. However, OH-PCBs loading directly to GC will lead to tailing chromatographic peaks and irreproducible peak areas [17] due to the interaction of -OH with basic sites in the injector and column. To overcome this weakness, OH-PCBs must be derivatized using different derivatization reagents, such as diazomethane [15,19], trimethylsilyl diazomethane [16], trifluoroacetyl [17], or N, O-bis (trimethylsilyl) trifluoroacetamide + trimethylchlorosilane (BSTFA + TMCS, 99:1)[18], etc., before GC analysis, whereas derivatization is time-consuming, and incomplete derivatization may introduce detection errors [20]. In recent years, the liquid chromatography–tandem mass spectrometry (LC–MS/MS) technique has been increasingly applied in the field of OH-PCBs detection on account of its direct injection without derivatization and fast chromatographic separation [13,21,22,23,24]. Nevertheless, there are more challenges to separating the OH-PCB congeners for the LC method than for the GC method since the resolution of shorter LC columns is usually lower than that of longer GC columns [20]. In addition, due to the low polarity and low ionization potential of OH-PCBs, LC–MS/MS method employed to determine these compounds frequently suffers from low sensitivity [25].

Before instrument analysis, the sample required extensive clean-up to remove the lipid and co-extracted materials. Generally, the extraction and clean-up procedure is labor-intensive, time-consuming, low throughput, and tedious, which includes liquid–liquid extraction and back extractions, sulfuric acid for lipid removal, pH partitioning and/or silica gel separation, multiple rotary evaporations, subsequent solvent dissolving and transfer [16,17,18,21,22]. Thus, a fast, simple, and reliable analytical method for determining these PCB metabolites needs to be highlighted. The current analytical methods reported for the determination of OH-PCBs are summarized in Table S1. Several new clean-up methods, such as online solid phase extraction [13,24] and molecularly imprinted solid phase extraction [23,25], have been developed for OH-PCBs detection. However, these methods are either only suitable for simple matrix samples (such as water, urine, and plasma) or still need complex procedures before the solid phase extraction treatment.

Therefore, the objective of this study was to develop a robust and sensitive LC–MS/MS method for the detection of the predominant penta- to hepta-chlorinated OH-PCBs in complex animal-derived food samples by using a more effortless and faster sample preparation procedure. The parameters affecting the purification, separation, ionization, and MS detection were investigated, and the analytical characteristics were studied in terms of linear range, limits of detection and quantification, accuracy, and precision. Finally, the proposed method was evaluated by the preliminary analysis of chicken eggs from laying hens exposed to a high level of PCB 101 (0.5 mg/kg in the feed) for two weeks.

## 2. Results and Discussion

### 2.1. Optimization of MS/MS Parameters

The main MS/MS parameters, including ion source mode, MRM transitions, and their collision energies, were optimized by infusing 1 μg/mL standard solution of the individual analytes at 10 μL/min directly into the mass spectrometer. Firstly, the positive and negative ESI modes were evaluated. The results showed that the precursor ion signals appeared only in ESI^-^ mode and could not be detected in positive ESI (ESI^+^) mode. Hence, the ESI^−^ mode was employed for the detection of OH-PCBs. This finding is in agreement with previous studies performed on LC–MS [20], LC–MS/MS [13], and liquid chromatograph-coupled to an ion trap/time-of-flight mass spectrometer LC–TOF–MS [25].

Secondly, the fragmentation patterns of OH-PCBs were investigated to select suitable MRM transitions. The MS scan spectra (*m*/*z*: 150–450) were acquired for each compound under the mass spectrometer conditions described in Section 2.4. The results revealed that OH-PCBs with the same number of chlorine atoms had similar mass spectrum patterns (not shown). As seen in Figure S1 Supplementary Material, there are three detectable fragment isotopic ion clusters for each analyte: a dominant deprotonated precursor ion [M-H]^−^ cluster, a minor product ion losing one HCl group [M-H-36]^-^ cluster, and a minimal product ion losing two HCl group [M-H-72]^−^ cluster. These fragmentation patterns of OH-PCBs matched with previous studies [17,22], which found the precursor ions of OH-PCBs were highly stable, resulting in a low response of the product ions. The present study showed that the relative abundance of the product ions of [M-H-36]^−^ and [M-H-72]^−^ were less than 30% and 5% of the precursor ion of [M-H]^−^, respectively. If we use the normal MRM method by monitoring two transitions generated from one precursor ion, which are from two product ions of losing one HCl group and two HCl groups, the detection sensitivities will be very low. In order to achieve high detection sensitivity, Zhai et al. (2013) employed the selected ion monitoring (SIM) method to monitor the precursor ions of OH-PCBs [20]. However, the SIM method is subjectable to being affected by matrix interference and has low accuracy. Due to the presence of chlorine atoms, a series of fragment isotopic cluster ions are produced in the mass spectra of OH-PCBs, which can be used as a choice for MRM transitions. For example, Quintanilla-Lopez et al. (2020) employed the two most abundant transitions (losing one HCl group) derived from the cleavage of corresponding isotopic ions of the quasi-molecular cluster for MRM detection [22]. This strategy makes both high sensitivity and high accuracy of the OH-PCBs detection available. The present study applied this methodology. Figure S1 showed that the first most abundant product ions of these OH-PCBs were [M+2-H-36]^−^ (*m*/*z*: 305), [M+2-H-36]^−^ (339), [M+4-H-36]^−^ (373), and the second most abundant product ions were [M-H-36]^−^ (303), [M-H-36]^−^ (337, almost the same with [M+4-H-36]^−^, 341), [M+6-H-36]^−^ (375) for penta-, hexa-, and hepta- chlorinated OH-PCBs, respectively, which have been selected as quantifier and qualifier ions for their detection.

**Figure 1:**
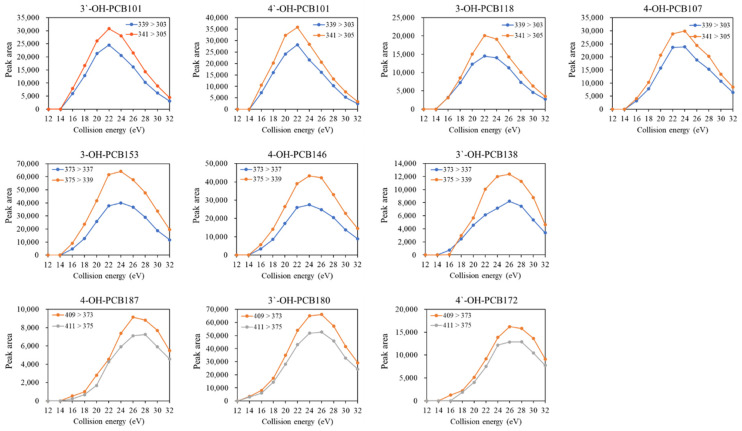
Effect of collision energy on the peak area of selected transitions of OH-PCB congeners.

Finally, the effect of collision energy on the response of each quantifier and qualifier transition was estimated. As shown in Figure 1, the peak areas of MRM transitions increased first to a narrow plateau. They then dramatically decreased with the increasing collision energy from12 eV to 32 eV, and the higher chlorination degree required slightly higher collision energy for obtaining the highest response of the transitions. The fragmentation behaviors obtained from the present study were similar to the findings reported by Quintanilla-Lopez et al. (2020) [22]. As a result, the optimum values of collision energy selected at the maximum of these curves were 22, 24, and 26 eV for penta-, hexa-, and hepta- OH-PCBs, respectively.

### 2.2. Optimization of LC Conditions

The LC conditions related to the chromatographic column, the mobile phase composition, and the modifier were optimized after the MS/MS conditions. The individual compound standard solutions and a full-mixed standard solution (20 ng/mL for each analyte) were applied to carry out the optimization of the LC parameters.

**Figure 2:**
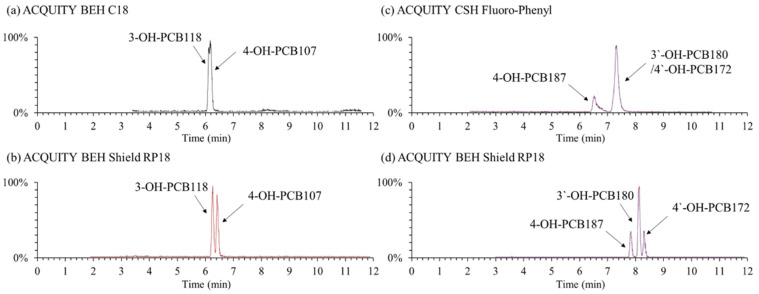
Example total ion chromatogram of typical OH-PCB congeners under the separation of different columns. Mobile phases were 0.1% formic acid-water (**a**) /0.1% formic acid-methanol (**b**) and the gradient elution program was 0–2 min, 25% (**a**); 2–10 min, 10% (**a**); 10–12 min, 2% (**a**); 12–13 min, 25% (**a**); 13–15 min, 25% (**a**).

Although some studies have been published for separating OH-PCBs using LC columns primarily based on octadecylsilane [21,23] or phenyl [13,24,25] stationary phases, it is still a complex problem to sufficiently separate dozens of OH-PCB isomers under the LC system. In the present study, three types of columns named ACQUITY BEH Shield RP18 (100 mm × 2.1 mm i.d., 1.7 µm particle size), ACQUITY BEH C18 (100 × 2.1, 1.7), and ACQUITY CSH Fluoro-Phenyl (100 × 2.1, 1.7) provided by Waters company (USA), were investigated for the separation of OH-PCB congeners. The first two are the octadecylsilane stationary phase, and the last one is the phenyl phase. It was found that the BEH Shield RP18 column had the best separation capability and got an acceptable separation for the selected OH-PCB congeners. In contrast, the regular C18 and phenyl-based columns could not sufficiently separate some specific congeners (Figure 2). For instance, the two hepta- OH-PCBs of 3′-OH-PCB180 and 4′-OH-PCB172 were not separated by the CSH Fluoro-Phenyl column at all. The same phenomenon was reported by Quinete et al. (2016), who employed another phenyl-based column of Kinetex PFP column (150 × 4.6 mm, 2.6 μm, Phenomenex, Torrance, CA, United States) [13]. The embedded carbamate group in the bonded phase ligand of the BEH Shield RP18 column provides alternate selectivity for phenolic compounds compared to straight-chain alkyl columns. It may be the reason why the BEH Shield RP18 column had a better separation ability for OH-PCB congeners than the normal C18 column. Quintanilla-Lopez et al. (2020) also reported an amide-type polar embedded C18 column HyPurity Advance (100 × 2.1 mm, 3 μm, Thermo Fisher Scientific, Waltham, MA, United States) was applied to separate eight penta- to hepta-chlorinated OH-PCBs satisfactorily [22]. Hence, we selected the BEH Shield RP18 column for the OH-PCBs separation.

**Figure 3:**
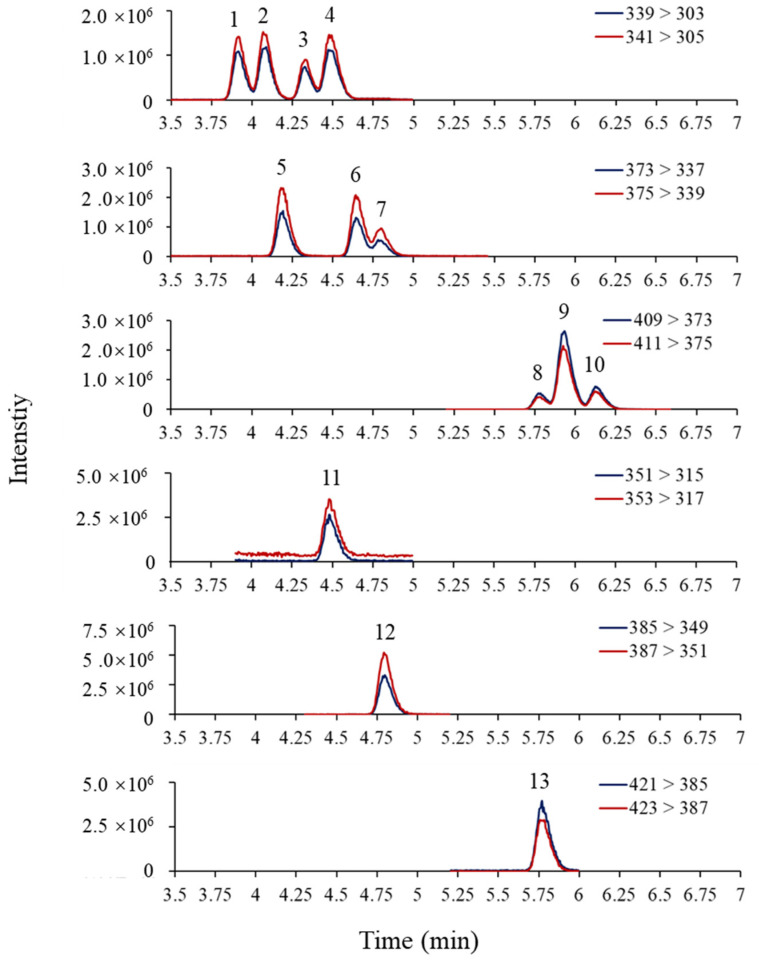
MRM chromatograms of OH-PCBs (10 ng/mL) and their ^13^C- labeled standards (20 ng/mL) under optimized instrumental conditions. 1: 3′-OH-PCB101, 2: 4′-OH-PCB101, 3: 3-OH-PCB118, 4: 4-OH-PCB107, 5: 3-OH-PCB153, 6: 4-OH-PCB146, 7: 3′-OH-PCB138, 8: 4-OH-PCB187, 9: 3′-OH-PCB180, 10: 4`-OH-PCB172, 11: ^13^C_12_-4-OH-PCB107, 12: ^13^C_12_-4-OH-PCB146, 13: ^13^C_12_-4-POH-CB187.

The mobile phase composition can significantly affect the separation and sensitivity of OH-PCBs detection by LC–MS/MS. This study tested two mobile phase compositions: (1) water–methanol containing 0.01% FA; and (2) water–acetonitrile containing 0.01% FA. The mobile phases based on water–acetonitrile showed a very poor peak separation (Figure S2). However, the mobile phases based on water–methanol showed a better peak separation, so it was selected for the present study. After choosing the mobile phase, FA and ammonium formate used as mobile phase modifiers were evaluated according to the peak shapes and peak signals. Firstly, the addition of 0%, 0.005%, 0.01%, and 0.1% of FA in the mobile phases was examined. The results revealed that increasing FA content in the mobile phases could improve the separation of OH-PCB peaks (Figure S3) but reduce the peak response (Figure S4A). The addition of 0.01% FA to the mobile phase achieved a balance between peak resolution and response then was therefore chosen as the final mobile phase composition. Ammonium formate as a modifier did not affect the peak shape (not shown), but it suppressed the peak signals as the concentration increased (Figure S4B). Therefore, ammonium formate was not introduced to the mobile phase. The MRM chromatograms of OH-PCBs and ^13^C- labeled standards were shown in Figure 3 under the optimized instrumental conditions.

### 2.3. Optimization of the Sample Preparation

**Figure 4:**
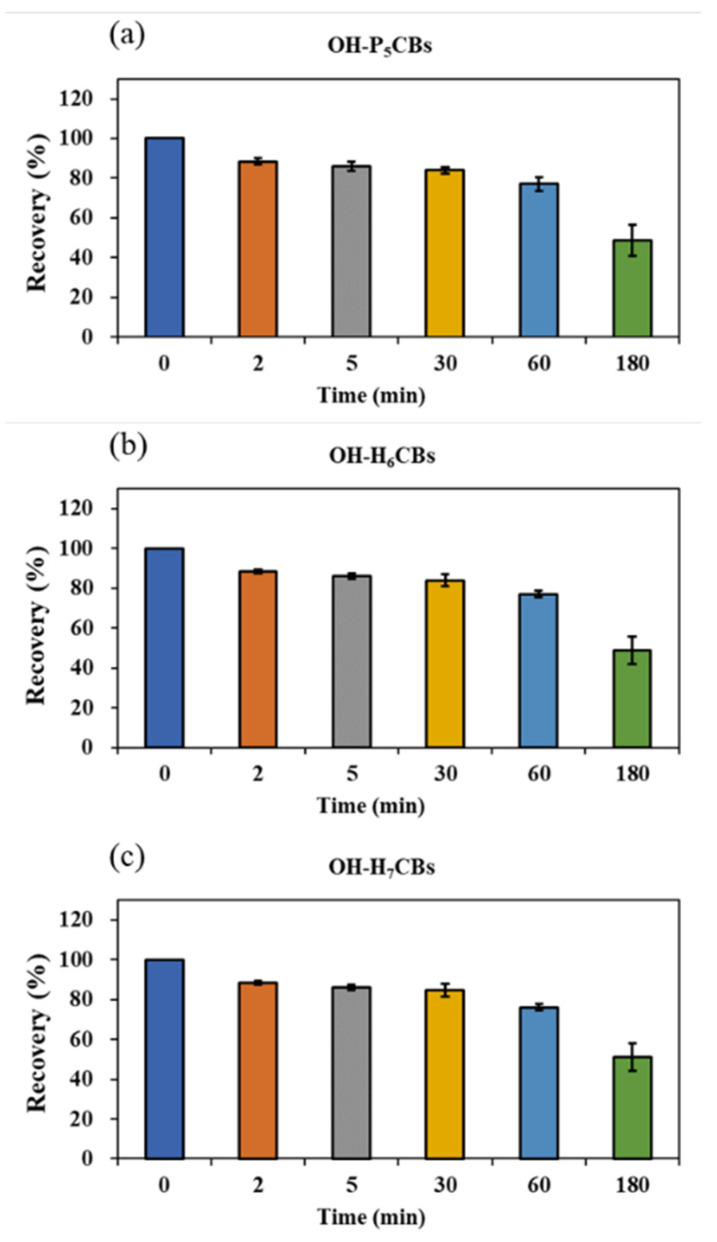
Mean recoveries of penta- (**a**), hexa- (**b**), and hepta-chlorinated (**c**) OH-PCBs treated with H_2_SO_4_ at different time points. Rectangles indicate the average recovery of the OH-PCBs containing the same chlorine atoms, which is calculated by the individual OH-PCB peak area under the specific time point with H_2_SO_4_ treatment dividing the peak area without H_2_SO_4_ treatment. Each test has three replicates. Short bars indicate standard deviations.

**Figure 5:**
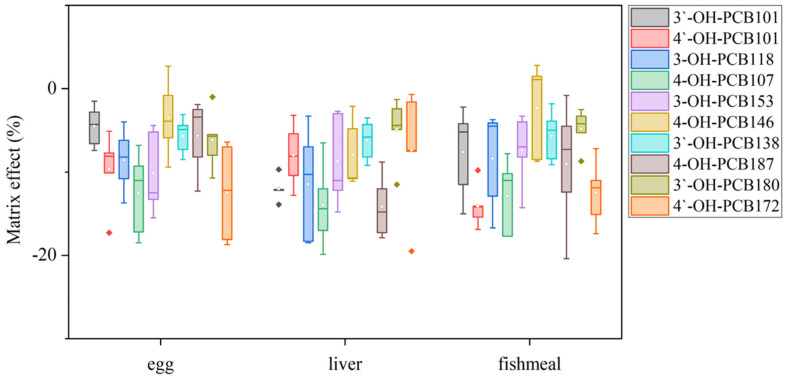
Box plots of matrix effects of the ten OH-PCBs in different sample matrices.

Since OH-PCBs are lipophilic compounds with a polar -OH group, one or a mixture of organic solvents such as dichloromethane, methyl t-butyl ether, *n*-hexane, methanol, and acetonitrile is often applied to extract OH-PCBs from various sample types [17,21,22,24,25]. The present study employed 50% n-hexane/DCM (*v/v*) with ultrasound to extract OH-PCBs from lyophilized animal-derived foods and achieved satisfactory recoveries (Table S2). Removing lipids and other co-extracted materials from complex matrix solutions is a significant challenge for OH-PCBs analyses. The pH partitioning is commonly reported as a decisive procedure for the OH-PCBs separation from neutral derivatives [16,21], but it is time-consuming and solvent-wasting, and a low recovery for higher chlorinated congeners is recognized [25]. H_2_SO_4_ treatment exists as an effective process for the removal of lipids and other organic compounds, thus widely employed in POPs analysis [26,27]. However, due to the limitation on its degradation of some phenolic compounds, this method uses less for analyzing the hydroxylated metabolites of POPs [16]. However, we found the penta- to hepta-chlorinated OH-PCBs were highly resistant to H_2_SO_4_ degradation. It was possibly due to the presence of multiple chlorine atoms in the biphenyl molecule blunting the reactive sites with H_2_SO_4_. As seen in Figure 4, when OH-PCBs were mixed with H_2_SO_4_ in the solution of n-hexane, the mean recoveries of these penta- to hepta-chlorinated OH-PCBs were higher than 80% within 30 min. Since the reaction time required for lipid removal by H_2_SO_4_ is generally less than 20 min, the treatment is acceptable for penta- to hepta-chlorinated OH-PCBs analysis. We further found that if the H_2_SO_4_ was directly mixed with the sample extract, the solution would become a slurry and the solid-liquid separation took more than 10 h (Figure S5a,b). In contrast, using 44% H_2_SO_4_-silica gel may successfully achieve lipid removal and solid-liquid separation within 30 min (Figure S5c). Hence, the H_2_SO_4_-silica gel was chosen as the purification method in this study. After the purification procedure, there are still non-polar compounds present in the solution that interfere with the detection of OH-PCBs by LC–MS/MS. To further separate OH-PCBs from non-polar interferences, a reported 5% hydrated silica gel column [16] was employed and generated reasonable yields of matrix effects (Figure 5) and recoveries (Table S2) of selected OH-PCBs.

### 2.4. Validation of the Proposed Method

The analytical performance parameters of this optimized LC–MS/MS method for the selected OH-PCBs are shown in Table 1. Linear calibration curves, based on internal standard-corrected response versus concentrations, were obtained in the range of 1.0–80.0 ng/mL with correlation coefficients (R^2^) ranging from 0.9912 to 0.9990. The calculated LODs ranged from 0.005 to 0.007 μg/kg for OH-P5CBs, 0.005 to 0.010 μg/kg for OH-H6CBs, and 0.003 to 0.010 μg/kg for OH-H7CBs, respectively. Compared with different instrumental methods reported for the determination of OH-PCBs in biological samples, the present method showed about 5–15-fold lower LOQs than that in plasma by LC–MS/MS [21], 200–700-fold lower LOQs than that in carp muscle by GC-ECD [18], and 3–5-fold higher LOQs than that in liver and blood by GC-MS/MS [16], respectively, which indicated the reasonable sensitivity was obtained in this study. As shown in Figure 5, matrix effects were almost negative values. It illustrated the suppressions of the analyte signal existed. The matrix effect mainly resulted from the sample clean-up procedure without the complete removal of the co-extracted matrix components. For a certain target analyte in the same matrix, a larger absolute value of the matrix effect indicates more co-extracted interferences retained in the solution. The conclusion of having no matrix effect would generally be drawn, with its value ranging from −20% to 20%[28]. In the present study, the matrix effects values were higher than −20% but lower than 3%, whose absolute values are far lower than that (ranging from −11.4 to −99.5%) obtained from the plasma matrix by the SPE clean-up method [24]. The results supported the sample preparation procedure showcased in this study for its effective removal of co-extracted matrix components. The resulting recoveries and RSD values are shown in Table S2. The average recoveries were between 76.7% and 116.5%, while the RSD values ranged from 2.2% to 18.4%. As shown in Figure S6, the values determined by this method were consistent with the ones confirmed using GC-MS/MS by the pH partitioning clean-up methods [14] with less than 15% relative deviations. Moreover, the present method has lower RSD values.

## 3. Material and Methods

### 3.1. Chemical and Reagents

Ten native penta- to hepta-chlorinated OH-PCBs (purity > 98%) were investigated in this study based on their occurrence in real samples and availability in the commercial approach. Both native and ^13^C- labeled standards were sourced from Wellington Laboratories (Guelph, Canada). Their chemical characteristics and abbreviations are summarized in Table 2. Mixed stock solutions for standards and ISs were prepared by combining suitable aliquots of various standard solutions to reach the concentration of 200 ng/mL of each compound and stored in a brown glass bottle at −18 °C. Series calibration solutions (native analyte: 1, 2, 5, 10, 20, 40, and 80 ng/mL; IS: 20 ng/mL) were prepared by taking 5, 10, 25, 50, 100, 200, and 400 μL of the mixed stock standards solution into individual centrifuge tubes and adding 50 μL of the mixed stock ISs solution to each tube, then drying them at room temperature with nitrogen, and finally dissolving the residue with 1 mL of 80% methanol/water (*v*/*v*).

Methanol, acetonitrile, ammonia, and formic acid of LC–MS grade were obtained from Thermo Fisher Scientific (Pittsburgh, PA, USA). The *n*-hexane and dichloromethane (DCM) of pesticide grade were purchased from J.T. Baker (Phillipsburg, NY, USA). Anhydrous sodium sulfate (Na_2_SO_4_, heated at 660 °C for 6 h before use) and concentrated sulfuric acid (H_2_SO_4_) of reagent grade (purity > 99.8%) were obtained from Sinopharm Chemical Reagent Co., Ltd. (Beijing, China). Silica gel (0.063–0.100 mm, activated at 550 °C for 12 h before use) was purchased from Merck (Darmstadt, Germany). Then, 44% H_2_SO_4_-silica gel was prepared by slowly dropping 44 g of H_2_SO_4_ to 66 g of activated silica gel and then shaking overnight, and 5% hydrated silica gel (5% H_2_O deactivated) was made by adding 5 g of H_2_O slowly to 95 g of activated silica gel and shaking overnight.

### 3.2. Instruments

Analysis of these 10 OH-PCBs was conducted using a UPLC system (Acquity) coupled with an MS/MS instrument (XEVO TQ-S, Waters, USA). Waters Masslynx software package (version 4.1, Waters, Milford, MA, USA) was used to control the instruments and to acquire and process data. Other laboratory equipment included a 3K15 high-speed refrigerated centrifuge (Sigma, St. Louis, MO, USA), a KQ-500DE ultrasound (Kunshan Instruments Company, China), an N-EVAP 112 nitrogen evaporator (Organomation, Berlin, MA, USA), and an HEI-VAP rotary evaporator (Heidolph, Germany).

### 3.3. Sample Preparation

All the samples were lyophilized, grounded, homogenized, and stored in sealed aluminum foil bags at −20 °C until analysis. After adding 10 μL of mixed ISs stock solution, each 5.0 g of sample was extracted twice by ultrasound with 30 mL of 50% *n*-hexane/DCM (*v*/*v*) for 30 min. After standing, the extract solution was transferred to a round-bottom flask and evaporated to dryness. The residue was re-dissolved in 100 mL n-hexane and mixed with 10–40 g of 44% H_2_SO_4_-silica gel on the basis of the fat load. The mixture was spun on the rotary evaporator at atmospheric pressure, 75 rpm, and 50 °C for approximately 20 min until the supernatant was clarified. Then, the supernatant was transferred to a flask while the solid residue was re-extracted twice with 30 mL of *n*-hexane. The combined solution was subsequently concentrated to 3–5 mL using the rotary evaporator at 450 mbar, 75 rpm, and 50 °C. A 5% hydrated silica gel column (diameter: 1.5 cm; the top-to-down material: 10 g of Na_2_SO_4_ and 3.0 g of 5% hydrated silica gel) was used to separate the target compounds. The column was preconditioned with 30 mL of *n*-hexane before loading samples. The concentrated sample solution was passed through the column by gravity, and the column was washed with 30 mL of *n*-hexane to remove the non-polar compounds (such as PCBs). Target analytes were eluted with 30 mL 40% *n*-hexane/DCM (*v/v*) into a pyriform flask and then evaporated to about 2.0 mL. The evaporator-concentrated solution was transferred into a Kuderna–Danish concentrator and dried with nitrogen. Finally, 50 μL of 80% methanol–water (*v*/*v*) was added to dissolve the target compounds for UPLC–MS/MS analysis.

### 3.4. UPLC–MS/MS Conditions

Chromatographic separation was performed using a BEH Shield RP18 column (100 mm × 2.1 mm i.d. and 1.7 µm particle size, Waters, USA) with water (A) and methanol (B) containing 0.01% formic acid (*v/v*) as the mobile phases. Gradient elution was employed, starting with 75% (by volume) B for 2 min, then increasing to 90% B over 8 min, and finally increasing to 98% B over a further 2 min before returning to 75% B over 1 min. This composition was held constant for 2 min before the next injection (for a total run time of 15 min). The column was kept at 40 °C, the mobile phase flow rate was 0.3 mL/min, and the injection volume was 5 μL.

The mass spectrometer was operated in the negative electrospray ionization (ESI^-^) mode with a capillary voltage of 2.0 kV, source offset of 60 V, source temperature of 150 °C, desolvation temperature of 450 °C, desolvation gas flow of 800 L/h, and cone gas flow of 150 L/h. Nitrogen (99.999%) was employed as the nebulizer, desolvation, and cone gas. Analyte detection was performed in the multiple reaction monitoring (MRM) mode using argon (99.999%) as the collision gas at a flow rate of 0.14 mL/min.

The specific transitions of precursor ion and product ion were as follows: 341 > 305 *m*/*z* (quantifier) and 339 > 303 (qualifier) for penta- OH-PCBs, 375 > 339 and 373 > 337 for hexa- OH-PCBs, 409 > 373 and 441 > 375 for hepta- OH-PCBs, 353 > 317 and 351 > 315 for ^13^C_12_-4-OH-PCB 107, 387 > 351 and 385 > 349 for ^13^C_12_-4-OH-PCB 146, 421 > 385 and 423 > 387 for ^13^C_12_-4-OH-PCB 187, with the optimized cone voltage of 30 V for all transitions and collision energy of 22, 24, and 26 ev for the transitions of penta-, hexa-, and hepta- OH-PCBs, respectively.

### 3.5. Method Validation

Thorough method validation was performed for four typical matrices of egg, liver (pig), and fishmeal. Three ISs were used as surrogates for the OH-PCBs with the same number of chlorines to compensate for the analyte loss during sample preparation and the matrix effects in these trials. The calibration curves with seven concentration levels (ranging from 1.0 to 80 ng/mL) for the analytes and fixed concentrations of the ISs (20 ng/mL) were generated. These curves were performed according to the ratios of the peak areas of the analytes to those of the IS compounds plotted against the analyte concentration. Matrix effects were calculated by comparing the analytical areas for extracted samples spiked at concentrations of 20 ng/mL with solvent standards, following the equation [22]:

(1) $$\mathrm{matrix}\mathrm{effects}=\left(\frac{\mathrm{peak}\mathrm{area}\mathrm{of}\mathrm{fortified}\mathrm{extract}}{\mathrm{peak}\mathrm{area}\mathrm{of}\mathrm{solvent}\mathrm{standard}}-1\right)\times 100\%$$

Accuracy and precision were expressed by determining values for recoveries and relative standard deviations (RSDs), using data after five replicate analyses towards blank solutions spiked with the analytes of 0.02, 0.2, or 0.4 μg/kg. The limit of detection (LOD) and limit of quantification (LOQ) values were calculated as the average concentration of each compound produced a signal-to-noise ratio (S/N) of 3 and 10, respectively, based on the sample at the lowest spiked concentration level. Finally, the proposed method was further verified using real egg samples, which were produced by laying hens after dietary exposure to PCB 101 (5 μg/kg in feeds) [14].

## 4. Conclusions

This study developed a UPLC–MS/MS method to simultaneously determine penta- to hepta-OH-PCBs in animal-derived food matrices, using the H_2_SO_4_-silica gel as the lipid remover followed by 5% hydrated silica gel separation in the process of pretreatment. Compared to other published methods, the sample preparation procedure displayed in this study is simple and time-saving and has low solvent consumption and efficient interference removal. The method performance parameters, including linearity, sensitivity, accuracy, and precision, have been verified to meet the needs of toxicological studies and food monitoring of OH-PCBs. Consequently, this analytical method will enable researchers to assess the characteristics of OH-PCBs in animal-derived food more comprehensively.

## Figures and Tables

**Table 1:** Analytical performance parameters.

Compound	Internal Standard	Calibration Curve	R^2^	LOD (μg/kg)	LOQ (μg/kg)
3′-OH-CB101	^13^C_12_-4-OH-PCB 107	Y = 1.88X–1.94	0.9960	0.005	0.015
4′-OH-CB101	^13^C_12_-4-OH-PCB 107	Y = 2.20X–2.63	0.9912	0.005	0.015
3-OH-CB118	^13^C_12_-4-OH-PCB 107	Y = 1.03X–1.12	0.9958	0.007	0.021
4-OH-CB107	^13^C_12_-4-OH-PCB 107	Y = 1.42X–1.25	0.9960	0.005	0.015
3-OH-CB153	^13^C_12_-4-OH-PCB 146	Y = 2.22X–1.63	0.9988	0.005	0.015
4-OH-CB146	^13^C_12_-4-OH-PCB 146	Y = 1.45X–1.08	0.9989	0.005	0.015
3′-OH-CB138	^13^C_12_-4-OH-PCB 146	Y = 0.45X–0.47	0.9941	0.010	0.030
4-OH-CB187	^13^C_12_-4-OH-PCB 187	Y = 0.39X–0.34	0.9966	0.010	0.030
3′-OH-CB180	^13^C_12_-4-OH-PCB 187	Y = 2.96X–2.14	0.9990	0.003	0.009
4′-OH-CB172	^13^C_12_-4-OH-PCB 187	Y = 0.72X–0.52	0.9988	0.010	0.030

**Table 2:** Characteristics of the selected OH-PCBs.

IUPAC Name	Abbreviation	CAS Number	M_mi_^a^	Log*K_OW_*^b^	Chemical Structure
2,2′,4,5,5′-Pentachloro-3-biphenylol	3′-OH-CB101	69278-58-6	339.9	6.50	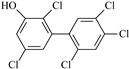
2,2′,4,5,5′-Pentachloro-4-biphenylol	4′-OH-CB101	Not available	339.9	Not available	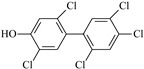
2,3′,4,4′,5-Pentachloro-3-biphenylol	3-OH-CB118	170946-11-9	339.9	Not available	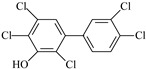
2,3,3′,4′,5-Pentachloro-4-biphenylol ^c^	4-OH-CB107	152969-11-4	339.9	6.50	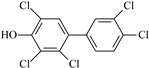
2,2′,4,4′,5,5′-Hexachloro-3-biphenylol	3-OH-CB153	54284-55-8	373.9	7.14	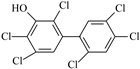
2,2′,3,4′,5,5′-Hexachloro-4-biphenylol ^c^	4-OH-CB146	145413-90-7	373.9	7.14	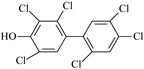
2,2′,3′,4,4′,5-Hexachloro-3-biphenylol	3′-OH-CB138	Not available	373.9	Not available	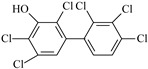
2,2′,3,4′,5,5′,6-Heptachloro-4-biphenylol ^c^	4-OH-CB187	158076-68-7	407.8	7.79	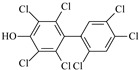
2,2′,3′,4,4′,5,5′-Heptachloro-3-biphenylol	3′-OH-CB180	158076-69-8	407.8	7.79	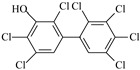
2,2′,3,3′,4′,5,5′-Heptachloro-4-biphenylol	4′-OH-CB172	158076-64-3	407.8	7.79	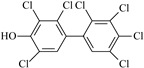

^a^ Monoisotopic mass. ^b^ EPISuite^TM^ predicted data. ^c^ Isotope labeled compounds, with twelve ^13^C atoms, of these standards have been used.

## Data Availability

Not applicable.

## Supplementary Materials

The following supporting information can be downloaded at: https://www.mdpi.com/article/10.3390/molecules27217247/s1, Table S1: Analytical methods reported for the determination of OH-PCBs; Table S2: Average recoveries and RSD for OH-PCBs; Figure S1: Mass spectra of representative OH-PCB congeners under MS scan model; Figure S2: Total ion chromatogram of selected OH-PCB congeners under the separation with 0.01% formic acid-water (A)/0.01% formic acid-acetonitrile (B) as mobile phase. The gradient elution program was 0–2 min, 25% (A); 2–10 min, 10% (A); 10–12 min, 2% (A); 12–13 min, 25% (A); 13–15 min, 25% (A); Figure S3: Example total ion chromatogram of 3 hepta- chlorinated OH-PCB congeners under the separation of water (A)/methanol (B) based mobile phase containing different concentrations of FA. The gradient elution program was 0–2 min, 25% (A); 2–10 min, 10% (A); 10–12 min, 2% (A); 12–13 min, 25% (A); 13–15 min, 25% (A); Figure S4: Effect of the addition of different concentrations of FA (a) and ammonium formate (b) to the mobile phase on the signal intensity of the OHPCB peaks. Rectangles indicate the average relative intensity of the OH-PCB peak areas, which is calculated by the individual OH-PCB peak area under the specific mobile phase dividing the peak area under the mobile phase containing 0.01% FA. Each test has five replicates. Short bars indicate standard deviations; Figure S5: Actual effect pictures of purifying egg sample extract with H_2_SO_4_ vs. H_2_SO_4_-silica gel. The H_2_SO_4_ introduced directly into the sample extract appeared as a slurry (a), and then the solid and liquid were separated after more than 10 h (b). The use of 44% H_2_SO_4_-silica gel successfully achieved lipid removal and solid-liquid separation within 30 min (c); Figure S6: Concentrations of 4′-OH-PCB 101 determined by the present method vs. the reported values performed on egg yolks. Rectangles indicate the average concentration of 4′-OH-PCB 101 (*n* = 3); short bars indicate standard deviations.
