# Supplementary material for: Development of an Ultra-Performance Liquid Chromatography-Tandem Mass Spectrometry Method for Hydroxylated Polychlorinated Biphenyls in Animal-Derived Food
Source: Molecules. 2022 Oct 25;27(21):7247. doi: 10.3390/molecules27217247 (PMC9656064; doi:10.3390/molecules27217247)
Supplement: Supplementary file 1 [file molecules-27-07247-s001.zip › molecules-1987184-supplementary.pdf]

Supplementary Materials

Development of an Ultra-performance Liquid Chromatography-tandem Mass Spectrometry Method for Hydroxylated Polychlorinated Biphenyls in Animal-derived Food

Wenyu Zhao [1], Na Cui [1], Yuan Huang [1], Zihao Wang [1], Peilong Wang [1], Haijun Liang [2], Ruiguo Wang *[a], Xiaoou Su *[a]

[1] *Institute of Quality Standards and Testing Technology for Agro-Products, Chinese Academy of Agricultural Sciences, Beijing 10081, China*

[2] *CHINA FEED Magazine Agency, Beijing 100710, China*

*Co-corresponding author: Ruiguo Wang

E-mail: wangruiguo@caas.cn

Tel: +86-10-80106576

*Co-corresponding author: Xiaoou Su

E-mail: suxiaoou@126.com

Tel: +86-10-80106307

Present address: No 12, southern street of Zhongguancun, Haidian District, Beijing, P.R.China, 100081

Table S1. Analytical methods reported for the determination of OH-PCBs.

Analytes	Sample type (size)	Sample preparation	Separation/detection	Detection limits	Recovery (%)	RSD (%)	Published year (Ref.)
11 bi- to hexa-chlorinated OH-PCBs	Egg (2 g)	Mixed with 25 g of Sodium sulfate, Soxhlet-extracted with 200 ml methylene chloride, LLE treatment with 50 ml of 0.25 M NaOH, the aqueous phase was acidic with sulfuric acid to pH < 2 and extracted three times with 100 ml methylene chloride, concentrated to 1 mL for derivatization	GC-MS/MS, MRM<br>DB-5 MS fused-silica capillary column, 60 m × 0.25 mm I.D. with a 0.25 mm bonded film;<br>Derivatization: trifluoroacetyl	Np	Np	Np	1997 ([1])
10 penta- to hepta-chlorinated OH-PCBs	Plasma (4-6 g wet weight)	acidification with HCI (6M, 1mL) and addition of 2-propanol (3 mL), extracted with MTBE/hexane (1:1, v:v), the organic extracts were partitioned with potassium hydroxide (1M in 50% ethanol), the alkaline phase was acidified and back-extracted into MTBE/hexane, the HO-PCBs were further isolated on a Florisil (1.2% w/w H_2O deactivated, 5 g) column and eluted with 60 mL of dichlormethane /hexane/methano] (50:45:5), dryness and reconstituted in 100 µL of 3:1 methanol/water	LC-ESI(-)-MS/MS, MRM<br>Finesse Genesis C18 (150-mm length, 2.1-mm i.d., 4-µm particles) column;<br>Mobile phase: methanol/water, gradient;<br>MRM: $[M-H-36]^-$<br>Quantification: internal calibration	LOQ: 50 pg g^{-1} (wet weight)	73-85	4-6	2005 ([2])
Chiral OH-PCBs (4-OH-PCB 91, 5-OH-PCB 91, 4-OH-PCB 95, 5-OH-	Rat liver microsomal	Np	HPLC-MS, ESI-, SIM;<br>Chiral column (Nucleodex β-PM);<br>Mobile phase:	iLOD: 0.30-0.60 ng mL^{-1}	82.3-96.5 for 4-OH-PCB 159	0.6-7.6	2013([3])

PCB 95, 5-OH-PCB 149)			water/acetonitrile, gradient;<br>SIM: [M+2-H]$^-$;<br>Quantification: external calibration				
53 tri- to octa-chlorinated OH-PCBs	Liver, brain (2.5 g wet weight)	Samples were denatured with 3mL of 6 M hydrochloric acid, adding 2-propanol (9mL), extracted with 50% methyl t-butyl ether/hexane by a homogenizer, the organic phase was dried and resolved with hexane, LLE treatment with 1 M potassium hydroxide in ethanol:water (1:1, v:v), the ethanolic phase was acidified and then extracted twice with 60 mL of 50% methyl t-butyl ether/hexane, followed by evaporation and redissolved with 50% methyl t-butyl ether/hexane, repeat LLE treatment, the organic phase was dried and redissolved with hexane, purified by passing a 5% H_2O deactivated silica gel column, eluting with 50% hexane/dichloromethane, dried and redissolved with 1 mL hexane; OH-PCBs were derivatized (methylation; overnight at 20℃) using methanol and trimethylsilyl diazomethane, then treated by GPC using a column packed with 50 g of Bio-Beads S-X3; then passed through 3 g of activated silica gel, eluted with 140 mL of 10% dichloromethane /hexane and	(A) GC/ECNI-MS<br>DB5-MSUI (40 m × 0.18 mm ID × 0.18 µm film)<br>SIM: [M]$^-$, [M+2]$^-$, [Cl]$^-$, [M-CH3]$^-$;<br><br>(B) GC-HRMS<br>DB-5MS (60 m × 0.25 mm ID × 0.25 µm film)<br>SIM: [M]$^+$, [M+2]$^+$, [M+2-COCH3]$^+$<br><br>Quantification: isotope dilution	(A)<br>LOD: 0.58-2.6 pg g^{-1} wet weiht;<br><br>(B)<br>LOD: 0.36-1.6 pg g^{-1} wet weiht;	(A)<br>64.7-117;<br><br>(B)<br>70.4-120	(A)<br>4.7-14;<br><br>(B)<br>2.3-12	2014 ([4])

		concentrated to near dryness, dissolved in up to 50 µL of decane for GC/MS analysis.					
5 bi- to penta-chlorinated OH-PCBs	Water (1 L)	Water samples passed through a SPE cartridge with DMIP sorbents, eluted with hexane:ethyl acetate (1:1, v:v), dired and dissolved in 100 µL of methanol for analysis	LC-ESI(-)-MS/MS<br>Sunfire C18 reversed-phase column (4.6 × 250 mm^2, 5 µm);<br>Mobile phase: water/methanol, gradient;<br>MRM: $[M-H-36]^-$ and $[M-H-72]^-$ for all of selected OH-PCBs;<br>Quantification: external calibration	LOD: 11-82 fM	89-110	< 11%	2015 ([5])
6 tri- to hepta- OH-PCBs	Liver, blood	Samples were grounded, extracted using methanol:chloroform (1:2) mixture, extracts were washed with 100 ml of de-ionized water, organic extract was re-dissolved in 50–100 ml of n-hexane and mixed with 10–50 ml of sulphuric acid, the organic extract was subsequently extracted three times with 50 ml of 1 M ethanolic (50%) KOH solution;<br>Then, two different separation methods were used to separate phenolic compounds: (i) molecularly imprinted (MIP) solid phase extraction cartridges, and (ii) alkaline solution	UPLC/TOF-MS<br>A non-porous (core–shell) 10 cm × 2.1 mm × 1.7 mm PFP column;<br>Mobile phase: water–methanol 90:10 (A) and methanol–water 90:10 (B), each with 4 mM of ammonium formate, gradient;<br>m/z values corresponding to the $(M–H)^-$ pseudo-molecular ion cluster	LOD: 0.7-15.4 pg g^{-1};<br>LOQ: 2.3-51.4 pg g^{-1};	(i) MIP: 64-80;<br>(ii) alkaline solution: 28-64	(i) MIP: 11.6-17.8;<br>(ii) alkaline solution: 10.5-18.0	2015 ([6])
16 mono- to hepta-chlorinated OH-PCBs	Plasma (100 µL)	Enzymatic hydrolysis;<br>Protein precipitation with methanol;<br>Online solid phase extraction by using a turboflow C18-XL column	LC- MS/MS, ESI-, MRM<br>Kinetex PFP (150 × 4.6 mm, 2.6 mm) column;<br>Mobile phase: water/methanol,	LOQ (established as the concentration 5 times higher than background noise):	71-134 at spiked levels of 0.1, 1, 5 ng mL^{-1} in the plasma	Intra-day: 2.1-15.3;<br>Inter-day: 5.4-15.5	2015 ([7])

		(0.5 × 50 mm)	gradient;<br>MRM: [M-H-36]$^-$ and [M-H-72]$^-$ for mono-OH-PCBs; [M-H-36]$^-$ and [M+2-H-36]$^-$ for di- to hexa- OH-PCBs; [M+2-H-36]$^-$ and [M+4-H-36]$^-$ for hepta-OH-PCBs;<br>Quantification: internal calibration	0.02-0.1 ng mL^{-1}, expect for 4-OH-PCB76 with 0.5 ng mL^{-1}			
20 mono- to hepta-chlorinated OH-PCBs	Urine (200 µL)	Enzymatic hydrolysis;<br>Protein precipitation with methanol;<br>Online solid phase extraction by using a turboflow C18-XL column (0.5 × 50 mm)	LC- MS/MS, ESI-, MRM<br>Kinetex PFP (150 × 4.6 mm, 2.6 mm) column;<br>Mobile phase: water/methanol, gradient;<br>MRM: [M-H-36]$^-$ and [M-H-72]$^-$ for mono-OH-PCBs; [M-H-36]$^-$ and [M+2-H-36]$^-$ for di- to hexa- OH-PCBs; [M+2-H-36]$^-$ and [M+4-H-36]$^-$ for hepta-OH-PCBs;<br>Quantification: internal calibration	LOQ: 0.01-0.19 ng mL^{-1}	79-125	Within-run: 2;<br>Between-run:17	2016 ([8])
8 OH-PCBs (3 penta-, 2 hexa-, and 3 hepta-)	Human serum	Protein precipitation with 1 mL of formic acid plus 50 µL of acetonitrile;<br>Extraction with 5 mL of hexane, LLE treatment with 1 M potassium hydroxide in ethanol:water (1:1, v:v), the ethanolic phase was acidified and then extracted with 4 mL of hexane, followed by evaporation and redissolved with 100 µL of methanol:water (75:25, v:v), the reconstituted solution	LC-MS/MS, ESI-, MRM;<br>Polar embedded LC column HyPurity Advance (100 × 2.1 mm, 3 µm, amide-type, Thermo Fisher);<br>Mobile phase: water/methanol/2.6 pH buffer of formic acid/ammonium formate 5 mM, gradient;<br>MRM: [M-H-36]$^-$ and [M+2-H-36]$^-$ for OH-penta-CBs; [M+2-H-36]$^-$ and [M+4-H-36]$^-$ for	LOQ: 0.39-1.81 pg mL^{-1} expect for 4-OH-PCB 108 (20.2 pg mL^{-1})	77-109 for the extract without dilution;<br>92-105 for the extract with dilution	Intra-day: 2.0-9.5;<br>Inter-day: 2.6-9.6	2020 ([9])

		diluted 10 times before LC-MS/MS analysis for reducing the matrix effects	OH-hexa-CBs and OH-hepta-CBs;<br>Quantification: internal calibration				
3-OH-PCB 101, 4-OH-PCB 101	Carp muscle (1.0 g wet weight)	Homogenized;<br>Acidified (pH 3.0) with 2 M HCl, ultrasonic extracted with 15 mL of n-hexane:dichloromethane (1:1, v:v), the upper organic phase was dried and re-dissolved in 10 mL of n-hexane:dichloromethane (1:1, v:v), 1 mL H_2SO_4 was added for lipid removal, the organic phase was dried and re-dissolved in 1 mL of n-hexane, loaded to a silica gel column and eluted with 8 mL of n-hexane:dichloromethane (1:1, v:v)	GC-μECD;<br>DB-17MS capillary column (30 m × 0.25 mm × 0.25 μm, Agilent);<br>Derivatization reagent: BSTFA-TMCS (100 μL)<br>Quantification: external calibration	LOD: 2.0 μg kg^{-1};<br>LOQ: 5.0 μg kg^{-1}	93.7-99.7	4.25-8.38	2022 ([10])

Abbreviations:

RSD: relative standard deviation;

Ref.: reference;

Np: not provided;

LOD: limit of detection;

LOQ: limit of quantification;

iLOD: instrumental limit of detection;

iLOQ: instrumental limit of quantification;

HPLC-MS: high-performance liquid chromatography-mass spectrometry;

SIM: selected ion monitoring;

ESI-: electrospray in negative ionization;

LC: liquid chromatography;

MS/MS: tandem mass spectrometry

LLE: liquid-liquid extraction;

GC: gas chromatography;

ECD: electro capture detection;

μECD: micro electro capture detection;

BSTFA-TMCS: N,O-bis (trimethylsilyl)-trimethylchlorosilane (99:1, v:v);

HCl: hydrochloric acid;

H_2SO_4: concentrated sulfuric acid;

GPC: gel permeation chromatography;

ECNI: electron capture negative ionization;

HRMS: high-resolution mass spectrometry;

DMIP: dummy molecularly imprinted polymer;

SPE: solid-phase extraction;

ISs: internal standards;

MTBE: methyl-tert-butyl ether;

UPLC: ultra-performance liquid chromatography;

TOF-MS: time-of-flight mass spectrometer;

BSTFA + TMCS: N, O-bis (trimethylsilyl) trifluoroacetamide + trimethylchlorosilane

PFP: Pentafluorophenyl

Table S2. Average recoveries and RSD for OH-PCBs.

Compound	Egg (μg/kg)			Liver (μg/kg)			Fishmeal (μg/kg)		
	0.02	0.2	0.4	0.02	0.2	0.4	0.02	0.2	0.4
3`-OH-CB101	100.9 [a] (10.5) [b]	99.5(6.9)	107.5(4.7)	96.4(15.6)	85.6(5.5)	88.5(6.4)	87.8(10)	104.7(8.2)	109.5(8.2)
4`-OH-CB101	97.8(12.8)	102.7(9.1)	108.1(4.9)	97.3(9.4)	106.3(13.2)	105(6.3)	87.2(6.1)	99.6(4.2)	106.1(9.9)
3-OH-CB118	101.7(9.7)	100.8(8.1)	102.4(4.1)	103.6(8.7)	101.5(9.0)	104.5(16.3)	99.2(15.6)	107.5(10.2)	116.1(5.5)
4-OH-CB107	104.1(8.6)	102.5(7.4)	99.2(4.8)	102.7(6.9)	94.3(6.3)	111.6(5.6)	95.5(1.7)	98.1(8.6)	92.5(7.2)
3-OH-CB153	96.7(11.7)	95.1(16.5)	94.1(2.4)	80.1(4.2)	93.2(4.1)	97.1(6.4)	106.8(10.8)	101.8(11.9)	89.2(10.1)
4-OH-CB146	110.1(5.6)	106.5(9.4)	104.2(5.1)	95.5(7.9)	84.5(7.6)	104.7(8.5)	108(5.5)	114.3(4.7)	102.2(4.4)
3`-OH-CB138	95.8(10.4)	83.9(13.9)	94.6(7.1)	81.4(16.4)	89.6(12.6)	90.7(12.8)	103.1(5.7)	97.7(10.5)	87.9(4.7)
4-OH-CB187	83.9(11.3)	77.8(9.4)	101.8(13.4)	90.8(7.7)	97.1(18.4)	87.9(15.1)	91.6(4.3)	86(11.4)	89.1(13.3)
3`-OH-CB180	102.9(15.6)	113.1(12.7)	100.8(12.2)	102.7(18.2)	86.1(17.7)	101(16.1)	91.9(4.1)	117.9(2.4)	108.3(3.4)
4`-OH-CB172	95.7(11.5)	90.4(10.2)	110.4(13.3)	77.8(5.2)	111.5(6.6)	86.6(17.9)	94.5(10.9)	101(14.5)	96.4(15.7)

[a] Average recovery (%).

[b] RSD (%).

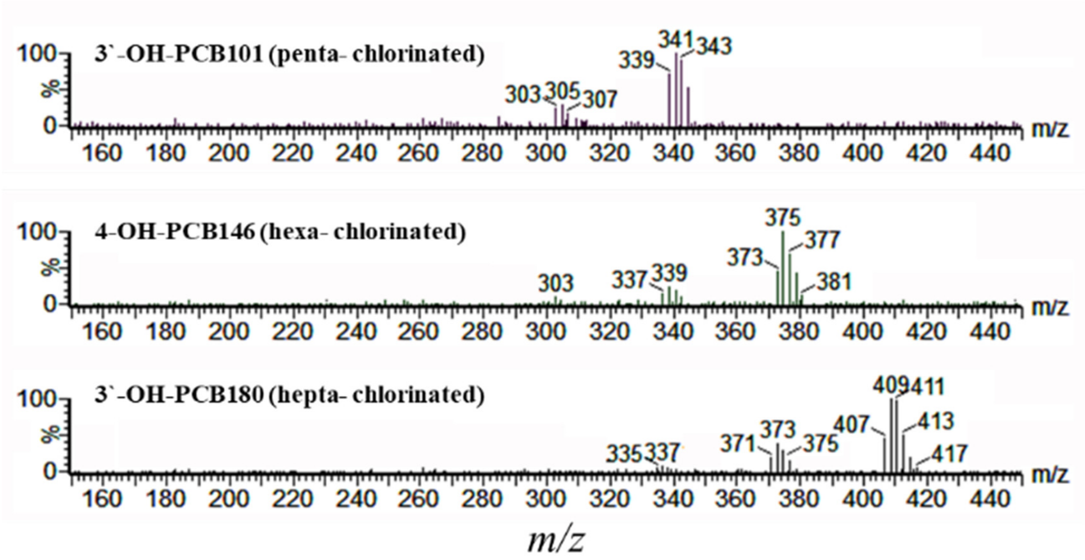

Figure S1. Mass spectra of representative OH-PCB congeners under MS scan model.

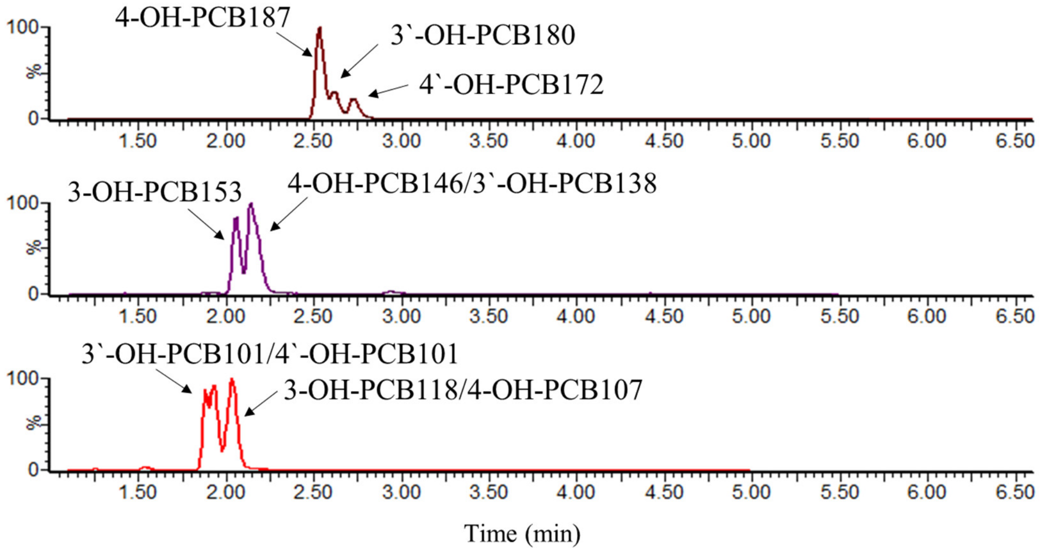

Figure S2. Total ion chromatogram of selected OH-PCB congeners under the separation with 0.01% formic acid-water (A)/0.01% formic acid-acetonitrile (B) as mobile phase. The gradient elution program was 0-2 min, 25% (A); 2-10 min, 10% (A); 10-12 min, 2% (A); 12-13 min, 25% (A); 13-15 min, 25% (A).

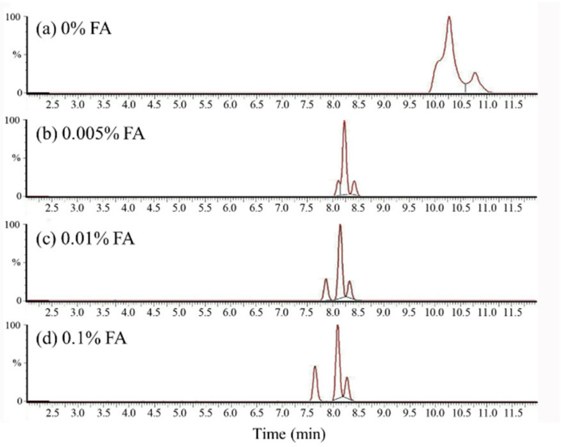

Figure S3. Example total ion chromatogram of 3 hepta- chlorinated OH-PCB congeners under the separation of water (A)/methanol (B) based mobile phase containing different concentrations of FA. The gradient elution program was 0-2 min, 25% (A); 2-10 min, 10% (A); 10-12 min, 2% (A); 12-13 min, 25% (A); 13-15 min, 25% (A).

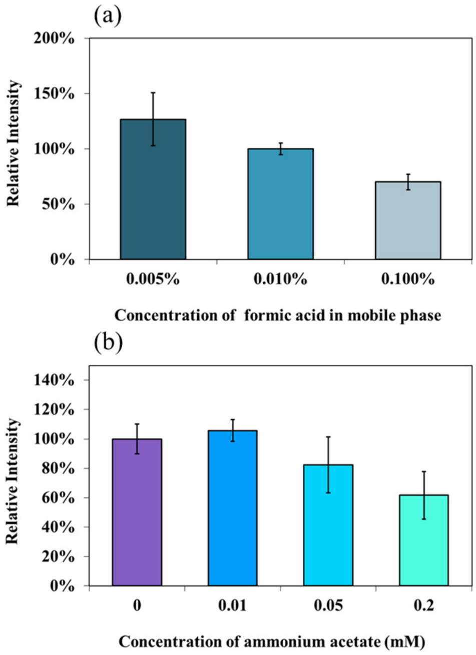

Figure S4. Effect of the addition of different concentrations of FA (a) and ammonium formate (b) to the mobile phase on the signal intensity of the OHPCB peaks. Rectangles indicate the average relative intensity of the OH-PCB peak areas, which is calculated by the individual OH-PCB peak area under the specific mobile phase dividing the peak area under the mobile phase containing 0.01% FA. Each test has five replicates. Short bars indicate standard deviations.

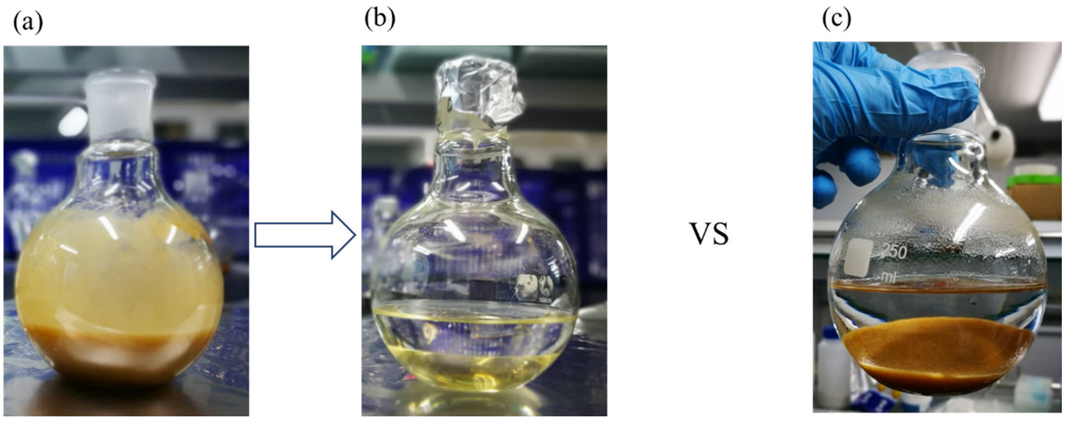

Figure S5. Actual effect pictures of purifying egg sample extract with H_2SO_4 VS H_2SO_4-silica gel. The H_2SO_4 introduced directly into the sample extract appeared as a slurry (a), and then the solid and liquid were separated after more than 10 h (b). The use of 44% H_2SO_4-silica gel successfully achieved lipid removal and solid-liquid separation within 30 min (c).

1

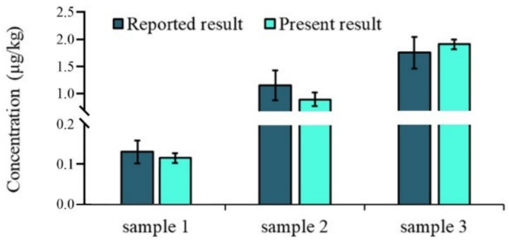

Figure S6. Concentrations of 4`-OH-PCB 101 determined by the present method VS the reported values performed on egg yolks. Rectangles indicate the average concentration of 4`-OH-PCB 101 (n = 3), short bars indicate standard deviations.

References

1. Abraham, V. M.; Jr, B. C. L., Determination of hydroxylated polychlorinated biphenyls by ion trap gas chromatography–tandem mass spectrometry. Journal of Chromatography A 1997, 790, (1–2), 131-141.
2. Letcher; R. J.; Li. H. X.; Chu. S. G., Determination of hydroxylated polychlorinated biphenyls (HO-PCBs) in blood plasma by high-performance liquid chromatography-electrospray ionization-tandem quadrupole mass spectrometry. *Journal of analytical toxicology* **2005,** 29, (4), 209-216.
3. Zhai, G. S.; Wu, X. N.; Lehmler, H. J.; Schnoor, J. L., Atropisomeric determination of chiral hydroxylated metabolites of polychlorinated biphenyls using HPLC-MS. *Chemistry Central Journal* **2013,** 7, (1), 1-9.
4. Eguchi, A.; Nomiyama, K.; Ochiai, M.; Mizukawa, H.; Nagano, Y.; Nakagawa, K.; Tanaka, K.; Miyagawa, H.; Tanabe, S., Simultaneous detection of multiple hydroxylated polychlorinated biphenyls from a complex tissue matrix using gas chromatography/isotope dilution mass spectrometry. *Talanta* **2014**, 118, 253-61.
5. Liang, R.; Zhao, Y.; Su, Y.; Qin, W., Determination of hydroxylated polychlorinated biphenyls by offline solid-phase extraction-liquid chromatography-tandem mass spectrometry using a molecularly imprinted polymer as a sorbent for sample preconcentration. *Talanta* **2015**, 144, 115-21.
6. Roszko, M.; Szymczyk, K.; Jedrzejczak, R., Simultaneous separation of chlorinated/brominated dioxins, polychlorinated biphenyls, polybrominated diphenyl ethers and their methoxylated derivatives from hydroxylated analogues on molecularly imprinted polymers prior to gas/liquid chromatography and mass spectrometry. *Talanta* **2015**, 144, 171-83.
7. Quinete, N.; Kraus, T.; Belov, V. N.; Aretz, C.; Esser, A.; Schettgen, T., Fast determination of hydroxylated polychlorinated biphenyls in human plasma by online solid phase extraction coupled to liquid chromatography-tandem mass spectrometry. *Anal Chim Acta* **2015**, 888, 94-102.
8. Quinete, N.; Esser, A.; Kraus, T.; Schettgen, T., Determination of hydroxylated polychlorinated biphenyls (OH-PCBs) in human urine in a highly occupationally exposed German cohort: New prospects for urinary biomarkers of PCB exposure. *Environ Int* **2016**, 97, 171-179.
9. Quintanilla-Lopez, J. E.; Galindo-Iranzo, P.; Lebron-Aguilar, R.; Gomara, B., Congener-specific determination of hydroxylated polychlorinated biphenyls by polar-embedded reversed-phase liquid chromatography-tandem mass spectrometry. *J Chromatogr A* **2020**, 1626, 461353.
10. Wang, S.; Huang, X. Y.; Wang, M. Y.; Tian, L. L.; Li, X. L.; Kong, C.; Han, F.; Lou, X. Y.; Ye, H. L.; Shi, Y. F., Simultaneous determination of polychlorinated biphenyl 101 (PCB101) and its hydroxylated, methoxylated and methyl sulfonated metabolites in aquatic organisms by solid-phase extraction (SPE) and gas chromatography-microelectron capture detection (GC-mu ECD). *Analytical Letters* **2022**, 55, (5), 784-795.
